# 2,3-Difluoro­benzoic acid

**DOI:** 10.1107/S1600536808001232

**Published:** 2008-01-18

**Authors:** Aleksandra A. Knapik, Wladek Minor, Maksymilian Chruszcz

**Affiliations:** a University of Virginia, Department of Molecular Physiology and Biological Physics, 1340 Jefferson Park Avenue, Charlottesville, VA 22908, USA

## Abstract

2,3-Difluoro­benzoic acid, C_7_H_4_F_2_O_2_, forms dimers that are stabilized by hydrogen bonds. The dimers are stacked and the stacks are held together by weak C—H⋯F and C—H⋯O inter­actions.

## Related literature

For related literature, see: Juhler & Mortensen (2002 ▶); Malone *et al.* (2006 ▶); Potrzebowski & Chruszcz (2007 ▶).
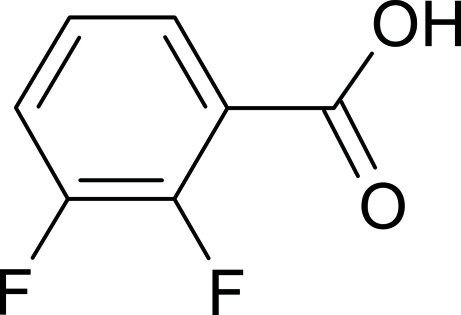


## Experimental

### 

#### Crystal data

C_7_H_4_F_2_O_2_
*M* *_r_* = 158.10
Monoclinic, 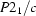

*a* = 3.761 (1) Å
*b* = 6.520 (1) Å
*c* = 26.521 (2) Å
β = 92.27 (1)°
*V* = 649.8 (2) Å^3^
*Z* = 4
Mo *K*α radiation
μ = 0.15 mm^−1^
*T* = 293 (2) K
0.15 × 0.15 × 0.02 mm

#### Data collection

Rigaku R-AXIS RAPID diffractometer
Absorption correction: multi-scan (Otwinowski *et al.*, 2003 ▶) *T*_min_ = 0.98, *T*_max_ = 1.00 (expected range = 0.977–0.997)
25713 measured reflections
1881 independent reflections
1371 reflections with *I* > 2σ(*I*)
*R*_int_ = 0.031

#### Refinement

*R*[*F* ^2^ > 2σ(*F* ^2^)] = 0.043
*wR*(*F* ^2^) = 0.133
*S* = 1.06
1881 reflections
116 parameters
All H-atom parameters refined
Δρ_max_ = 0.28 e Å^−3^
Δρ_min_ = −0.13 e Å^−3^

### 

Data collection: *HKL-2000* (Otwinowski & Minor, 1997 ▶); cell refinement: *HKL-2000*; data reduction: *HKL-2000*; program(s) used to solve structure: *SHELXS97* (Sheldrick, 2008 ▶) and *HKL-3000SM* (Minor *et al.*, 2006 ▶); program(s) used to refine structure: *SHELXL97* (Sheldrick, 2008 ▶) and *HKL-3000SM*; molecular graphics: *HKL-3000SM*, *Mercury* (Macrae *et al.*, 2006 ▶), *ORTEPIII* (Burnett & Johnson, 1996 ▶) and *ORTEP-3* (Farrugia, 1997 ▶); software used to prepare material for publication: *HKL-3000SM*.

## Supplementary Material

Crystal structure: contains datablocks I, global. DOI: 10.1107/S1600536808001232/om2199sup1.cif

Structure factors: contains datablocks I. DOI: 10.1107/S1600536808001232/om2199Isup2.hkl

Additional supplementary materials:  crystallographic information; 3D view; checkCIF report

## Figures and Tables

**Table 1:** Hydrogen-bond geometry (Å, °)

*D*—H⋯*A*	*D*—H	H⋯*A*	*D*⋯*A*	*D*—H⋯*A*
C5—H5⋯O1^i^	0.94 (2)	2.65 (2)	3.509 (2)	153 (2)
O2—H2⋯O1^ii^	1.03 (3)	1.60 (3)	2.625 (2)	173 (3)
C6—H6⋯O2^iii^	0.95 (2)	2.67 (2)	3.498 (2)	146 (1)
C4—H4⋯F2^iv^	0.95 (2)	2.65 (2)	3.513 (2)	151 (2)

Symmetry codes: (i) 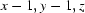; (ii) 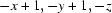; (iii) 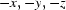; (iv) 
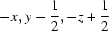
.

**Fig. 1.**
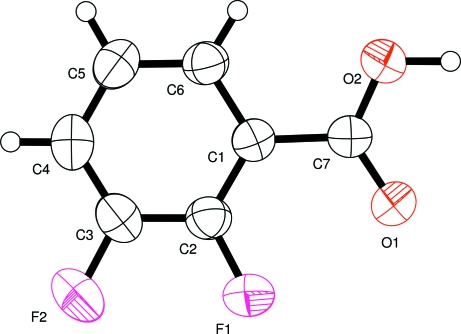
An asymmetric unit of 2,3-difluorobenzoic acid. Displacement ellipsoids are drawn at the 50% probability level, while hydrogen atoms are drawn as spheres of an arbitrary radius.

**Fig. 2.**
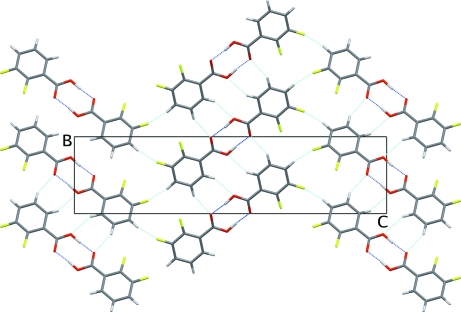
The packing of 2,3-difluorobenzoic acid shown along [010]. Hydrogen bonds are marked with blue, dashed lines. Weak C—H···F and C—H···O interactions are shown as light-blue, dashed lines.

## supplementary crystallographic information

### Comment

2,3-Difluorobenzoic acid (I) (Fig. 1) is used as a tracer for determining the extent of recovery of materials injected into oil wells (Malone *et al.*, 2006) or for observing water movment in soil (Juhler & Mortensen, 2002). 2,3-Difluorobenzoic acid crystallized, like 3,5-difluorobenzoic acid (Potrzebowski & Chruszcz, 2007), in the space group *P*2_1_/*c* with one molecule per asymmetric unit. Both (I) and 3,5-difluorobenzoic acid form dimers in the crystal lattice (Fig. 2), but the dimers of the two compounds pack differently. The molecules of (I) pack more tightly in the crystal, as the crystal density is 8% higher than in case of 3,5-difluorobenzoic acid. The dimers of (I) are stabilized by hydrogen bonds (Table 1). The dimers are arranged in stacks that are held together by weak C—H···F and C—H···O interactions (Fig. 2).

### Experimental

2,3-Difluorobenzoic acid (98%) was purchased from Aldrich, and dissolved in 1-propanol. Single crystals suitable for X-ray diffraction study were obtained by slow evaporation at 293 K.

### Refinement

All hydrogen atoms were localized using the difference density Fourier map. Their positions and isotropic displacement parameters were refined.

### Crystal data

**Table:**

C_7_H_4_F_2_O_2_	*F*_000_ = 320
*M**_r_* = 158.10	*D*_x_ = 1.616 Mg m^−^^3^
Monoclinic, *P*2_1_/*c*	Mo *K*α radiation λ = 0.71074 Å
Hall symbol: -P 2ybc	Cell parameters from 25713 reflections
*a* = 3.761 (1) Å	θ = 3.1–30.0º
*b* = 6.520 (1) Å	µ = 0.15 mm^−^^1^
*c* = 26.521 (2) Å	*T* = 293 (2) K
β = 92.27 (1)º	Plate, colorless
*V* = 649.8 (2) Å^3^	0.15 × 0.15 × 0.02 mm
*Z* = 4	

### Data collection

**Table:**

Rigaku R-AXIS RAPID diffractometer	1881 independent reflections
Radiation source: fine-focus sealed tube	1371 reflections with *I* > 2σ(*I*)
Monochromator: graphite	*R*_int_ = 0.031
Detector resolution: 10 pixels mm^-1^	θ_max_ = 30.0º
*T* = 293(2) K	θ_min_ = 3.1º
ω scans with χ offset	*h* = −5→5
Absorption correction: multi-scan(Otwinowski *et al.*, 2003)	*k* = −9→9
*T*_min_ = 0.98, *T*_max_ = 1.00	*l* = −37→37
25713 measured reflections	

### Refinement

**Table:**

Refinement on *F*^2^	Secondary atom site location: difference Fourier map
Least-squares matrix: full	Hydrogen site location: difference Fourier map
*R*[*F*^2^ > 2σ(*F*^2^)] = 0.044	All H-atom parameters refined
*wR*(*F*^2^) = 0.133	*w* = 1/[σ^2^(*F*_o_^2^) + (0.0665*P*)^2^ + 0.0891*P*] where *P* = (*F*_o_^2^ + 2*F*_c_^2^)/3
*S* = 1.06	(Δ/σ)_max_ < 0.001
1881 reflections	Δρ_max_ = 0.29 e Å^−^^3^
116 parameters	Δρ_min_ = −0.13 e Å^−^^3^
Primary atom site location: structure-invariant direct methods	Extinction correction: none

### Special details

**Table:**

Geometry. All e.s.d.'s (except the e.s.d. in the dihedral angle between two l.s. planes) are estimated using the full covariance matrix. The cell e.s.d.'s are taken into account individually in the estimation of e.s.d.'s in distances, angles and torsion angles; correlations between e.s.d.'s in cell parameters are only used when they are defined by crystal symmetry. An approximate (isotropic) treatment of cell e.s.d.'s is used for estimating e.s.d.'s involving l.s. planes.
Refinement. Refinement of *F*^2^ against ALL reflections. The weighted *R*-factor *wR* and goodness of fit *S* are based on *F*^2^, conventional *R*-factors *R* are based on *F*, with *F* set to zero for negative *F*^2^. The threshold expression of *F*^2^ > σ(*F*^2^) is used only for calculating *R*-factors(gt) *etc*. and is not relevant to the choice of reflections for refinement. *R*-factors based on *F*^2^ are statistically about twice as large as those based on *F*, and *R*- factors based on ALL data will be even larger.

### Fractional atomic coordinates and isotropic or equivalent isotropic displacement parameters (Å^2^)

**Table:**

	*x*	*y*	*z*	*U*_iso_*/*U*_eq_	
C1	0.3028 (3)	0.18896 (18)	0.09787 (4)	0.0453 (3)	
C2	0.3710 (3)	0.2435 (2)	0.14793 (5)	0.0507 (3)	
C3	0.2895 (4)	0.1088 (2)	0.18600 (5)	0.0580 (3)	
C4	0.1428 (4)	−0.0797 (2)	0.17592 (6)	0.0607 (4)	
C5	0.0737 (4)	−0.1357 (2)	0.12626 (6)	0.0587 (3)	
C6	0.1521 (3)	−0.0033 (2)	0.08785 (5)	0.0517 (3)	
C7	0.3892 (3)	0.32666 (19)	0.05550 (5)	0.0485 (3)	
F1	0.5159 (3)	0.42443 (14)	0.16134 (3)	0.0748 (3)	
F2	0.3611 (3)	0.16829 (19)	0.23412 (3)	0.0893 (4)	
O1	0.5592 (3)	0.48801 (16)	0.06313 (4)	0.0673 (3)	
O2	0.2818 (3)	0.26822 (19)	0.01143 (4)	0.0742 (4)	
H2	0.330 (8)	0.372 (4)	−0.0169 (13)	0.158 (11)*	
H4	0.090 (5)	−0.174 (3)	0.2020 (8)	0.087 (6)*	
H5	−0.026 (5)	−0.264 (3)	0.1183 (7)	0.079 (5)*	
H6	0.104 (4)	−0.037 (3)	0.0534 (6)	0.057 (4)*	

### Atomic displacement parameters (Å^2^)

**Table:**

	*U*^11^	*U*^22^	*U*^33^	*U*^12^	*U*^13^	*U*^23^
C1	0.0441 (6)	0.0446 (6)	0.0472 (6)	0.0012 (5)	0.0014 (4)	−0.0005 (5)
C2	0.0550 (7)	0.0465 (6)	0.0503 (6)	0.0009 (5)	0.0003 (5)	−0.0037 (5)
C3	0.0635 (8)	0.0653 (8)	0.0451 (6)	0.0066 (6)	0.0024 (5)	0.0018 (6)
C4	0.0593 (7)	0.0625 (8)	0.0607 (8)	0.0018 (6)	0.0074 (6)	0.0151 (6)
C5	0.0565 (7)	0.0494 (7)	0.0699 (9)	−0.0047 (6)	0.0003 (6)	0.0058 (6)
C6	0.0524 (7)	0.0495 (7)	0.0529 (7)	−0.0036 (5)	−0.0017 (5)	−0.0021 (5)
C7	0.0503 (6)	0.0481 (6)	0.0471 (6)	−0.0027 (5)	0.0001 (5)	−0.0024 (5)
F1	0.1091 (7)	0.0572 (5)	0.0576 (5)	−0.0162 (5)	−0.0039 (5)	−0.0095 (4)
F2	0.1270 (9)	0.0956 (8)	0.0449 (5)	−0.0067 (7)	0.0015 (5)	−0.0011 (5)
O1	0.0901 (7)	0.0569 (6)	0.0544 (5)	−0.0240 (5)	−0.0021 (5)	0.0016 (4)
O2	0.1028 (9)	0.0728 (7)	0.0461 (5)	−0.0317 (6)	−0.0058 (5)	0.0013 (5)

### Geometric parameters (Å, °)

**Table:**

C1—C2	1.3885 (17)	C4—H4	0.95 (2)
C1—C6	1.3968 (17)	C5—C6	1.376 (2)
C1—C7	1.4842 (17)	C5—H5	0.94 (2)
C2—F1	1.3415 (16)	C6—H6	0.950 (16)
C2—C3	1.3819 (19)	C7—O1	1.2435 (16)
C3—F2	1.3508 (16)	C7—O2	1.2796 (15)
C3—C4	1.369 (2)	O2—H2	1.03 (3)
C4—C5	1.381 (2)		
			
C2—C1—C6	118.03 (12)	C5—C4—H4	119.0 (12)
C2—C1—C7	122.09 (11)	C6—C5—C4	120.17 (14)
C6—C1—C7	119.88 (11)	C6—C5—H5	119.2 (12)
F1—C2—C3	117.71 (12)	C4—C5—H5	120.6 (11)
F1—C2—C1	122.43 (12)	C5—C6—C1	121.28 (13)
C3—C2—C1	119.86 (12)	C5—C6—H6	122.0 (10)
F2—C3—C4	120.41 (13)	C1—C6—H6	116.8 (10)
F2—C3—C2	117.76 (14)	O1—C7—O2	122.84 (12)
C4—C3—C2	121.82 (13)	O1—C7—C1	121.00 (11)
C3—C4—C5	118.84 (13)	O2—C7—C1	116.15 (11)
C3—C4—H4	122.2 (12)	C7—O2—H2	114.3 (16)
			
C6—C1—C2—F1	179.84 (11)	C2—C3—C4—C5	−0.2 (2)
C7—C1—C2—F1	0.6 (2)	C3—C4—C5—C6	0.0 (2)
C6—C1—C2—C3	−0.03 (19)	C4—C5—C6—C1	0.2 (2)
C7—C1—C2—C3	−179.23 (12)	C2—C1—C6—C5	−0.17 (19)
F1—C2—C3—F2	0.0 (2)	C7—C1—C6—C5	179.05 (12)
C1—C2—C3—F2	179.88 (12)	C2—C1—C7—O1	7.3 (2)
F1—C2—C3—C4	−179.64 (13)	C6—C1—C7—O1	−171.85 (12)
C1—C2—C3—C4	0.2 (2)	C2—C1—C7—O2	−173.28 (12)
F2—C3—C4—C5	−179.87 (13)	C6—C1—C7—O2	7.53 (18)

### Hydrogen-bond geometry (Å, °)

**Table:**

*D*—H···*A*	*D*—H	H···*A*	*D*···*A*	*D*—H···*A*
C5—H5···O1^i^	0.94 (2)	2.65 (2)	3.509 (2)	153 (2)
O2—H2···O1^ii^	1.03 (3)	1.60 (3)	2.625 (2)	173 (3)
C6—H6···O2^iii^	0.95 (2)	2.67 (2)	3.498 (2)	146 (1)
C4—H4···F2^iv^	0.95 (2)	2.65 (2)	3.513 (2)	151 (2)

Symmetry codes: (i) *x*−1, *y*−1, *z*; (ii) −*x*+1, −*y*+1, −*z*; (iii) −*x*, −*y*, −*z*; (iv) −*x*, *y*−1/2, −*z*+1/2.
